# Supplementary material for: Antibody Titer Kinetics and SARS-CoV-2 Infections Six Months after Administration with the BNT162b2 Vaccine
Source: Vaccines (Basel). 2021 Nov 19;9(11):1357. doi: 10.3390/vaccines9111357 (PMC8620373; doi:10.3390/vaccines9111357)
Supplement: Supplementary file 1 [file vaccines-09-01357-s001.zip › vaccines-1434068-SI.pdf]

Table S1. Demographic characteristics, serological results and COVID-19 related information of the 12 HCPs post-vaccination infected by SARS-CoV-2.

Subject	Sex	Age (years)	Anti-RBD (BAU/mL)		Vaccination to infection (days)[a]	Symptoms	Close contacts[d]
			T_2	T_3			
1	M	76	2122	>2500	64	Asymptomatic	Yes
2	F	42	>2500	>2500	59	Asymptomatic	Yes
3	M	54	196	897	69	Partial anosmia/ ageusia	Yes
4	M	39	1866	>2500	N/A [b]	Asymptomatic	No
5	F	57	2047	>2500	N/A [b]	Asymptomatic	No
6	F	55	>2500	>2500	<0[c]	Asymptomatic	No
7	F	49	>2500	>2500	67	Partial anosmia/ageusia, cold, generalized pain	Yes
8	M	53	339	>2500	84	Asymptomatic	No
9	F	42	1131	>2500	42	Asymptomatic	Yes
10	F	55	2046	>2500	99	Partial anosmia/ageusia, cold	Yes
11	F	42	>2500	2495	14	Partial anosmia/ageusia, cold	Yes
12	F	56	1066	714	7	Asymptomatic	Yes

[a]Intervals are calculated from the day of the 2nd dose to the day of the first positive RT-PCR test.

[b]COVID-19 was asymptomatic and the HCPs found out about the infection only through the serological test at T_3

[c]Positivity was discovered by an occasional anti-N test performed at T_1

[d]"Close contacts" refers to the presence of a SARS-CoV-2 positive unvaccinated household at the time of infection.

Table S2. Antibody titers at T_0, T_1, T_2 and T_3 of the 48 HCPs selected for neutralization activity tests. Time intervals between blood withdrawal and the first vaccine dose are reported as well as Seronegativity/positivity at T_0. Data have been color coded according to the anti-RBD titer at T_1 (dark blue, >2500 U/mL; blue, 2500 - 500 U/mL; light blue, 500 – 50 U/mL; dark grey 50 - 10 U/mL; light gray 10 - 1 U/mL, white < 1 U/mL).

N°	COVID-19	Test results (U/mL)				Time intervals (days)		
		T0	T1	T2	T3	T0-T1	T0-T2	T0-T3
1	positive	686	19280	52450	7950	22	43	177
2	positive	59.8	26440	21550	3630	21	46	185
3	positive	4.61	3708	8600	965	21	43	187
4	positive	17.1	22200	25250	2048	21	43	187
5	positive	26.5	10480	70900	5470	22	43	186
6	positive	6.46	13000	10700	1217	21	42	175
7	positive	108	89880	67900	17760	20	42	187
8	positive	72.9	11960	20800	2213	21	42	172
9	positive	58.3	1621	5600	1308	21	42	187
10	negative	0	1282	7060	290	22	43	188
11	negative	0	972	3160	212	22	40	183
12	negative	0	810	6760	1036	21	42	189
13	negative	0	749	4220	591	22	43	186
14	negative	0	702	3820	513	22	43	187
15	positive	0	673	3500	590	21	42	179
16	negative	0	610	8820	1693	21	41	187
17	negative	0	455	7660	1372	21	42	190
18	negative	0	314	10680	3750	22	43	187
19	negative	0	270	3720	1485	21	41	175
20	negative	0	212	6420	2447	21	41	178
21	negative	0	179	2493	1579	21	46	177
22	negative	0	131	6980	1757	21	42	175
23	negative	0	90.7	1467	437	21	42	178
24	negative	0	60.7	4280	1379	21	42	179
25	negative	0	48.7	1357	380	21	43	183
26	negative	0	42.5	3800	1022	21	42	187
27	negative	0	36.2	2480	1206	21	41	185
28	negative	0	30.3	829	361	21	42	187
29	negative	0	25.5	893	701	21	42	172
30	negative	0	20	4600	715	21	45	180
31	negative	0	15.3	452	332	22	43	177

32	negative	0	11.4	1715	825	23	44	183
33	negative	0	9.74	1326	942	21	42	186
34	negative	0	9.64	1357	328	23	45	185
35	negative	0	8.14	639	417	22	43	187
36	negative	0	6.88	671	188	21	43	190
37	negative	0	5.7	567	75.9	21	42	177
38	negative	0	4.51	701	187	21	42	189
39	negative	0	3.22	365	220	21	42	175
40	negative	0	2.05	1427	265	22	43	188
41	negative	0	0.99	386	409	20	41	176
42	negative	0	0.68	375	199	21	41	175
43	negative	0	0.57	483	226	21	39	180
44	negative	0	0.44	110	5160	21	46	183
45	negative	0	0.4	21.8	24.7	21	42	187
46	negative	0	0.4	66.5	169	22	40	185
47	negative	0	0.4	257	167	21	42	172
48	negative	0	0.4	0.4	9.1	21	42	182

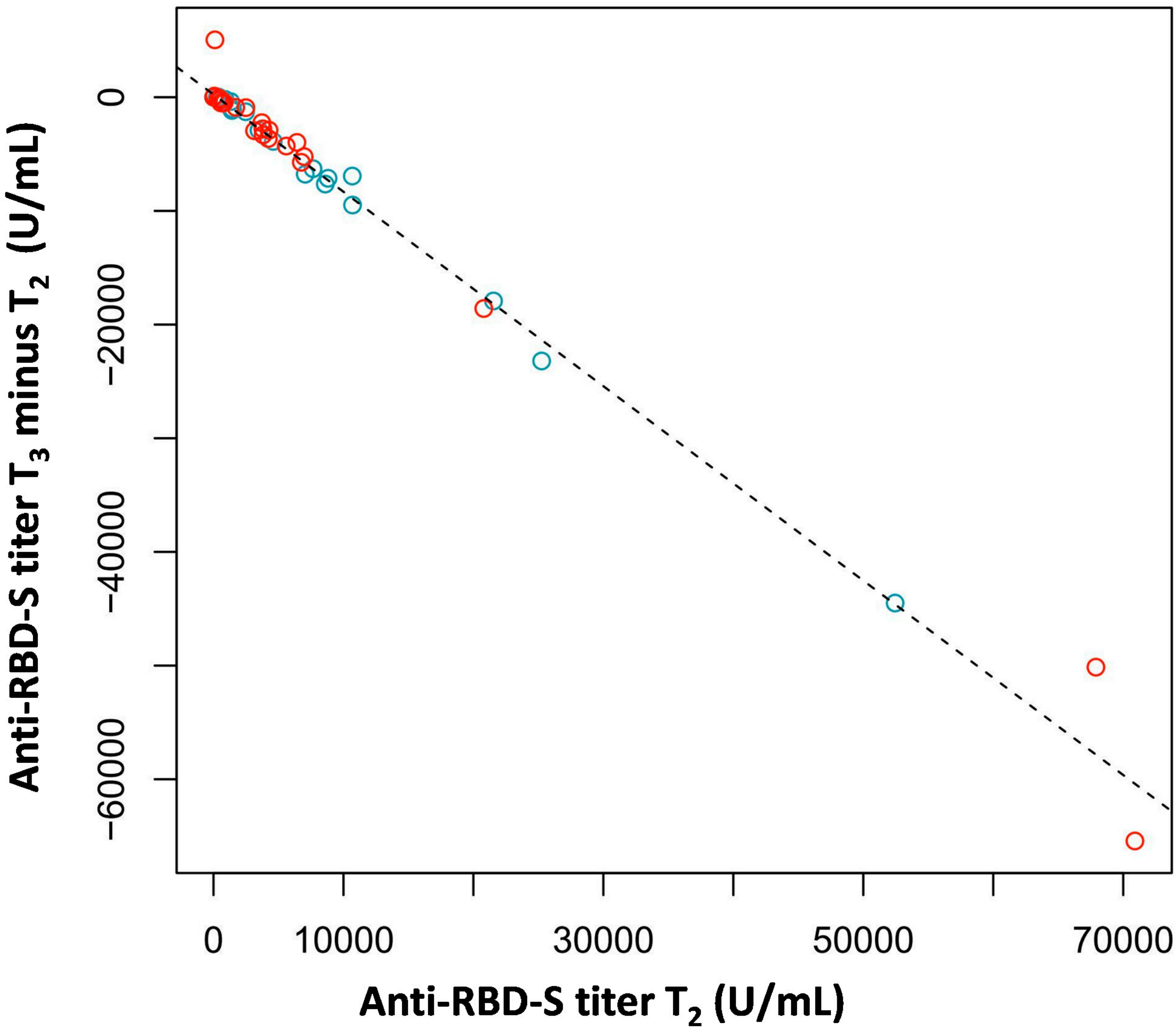

Figure S1. Association between antibody titer peak at T_2 versus T_3 minus T_2 variation in the 48 samples described in section 3.7. Dashed line represents the robust linear regression analysis. Red dots: females; blue dots: males.

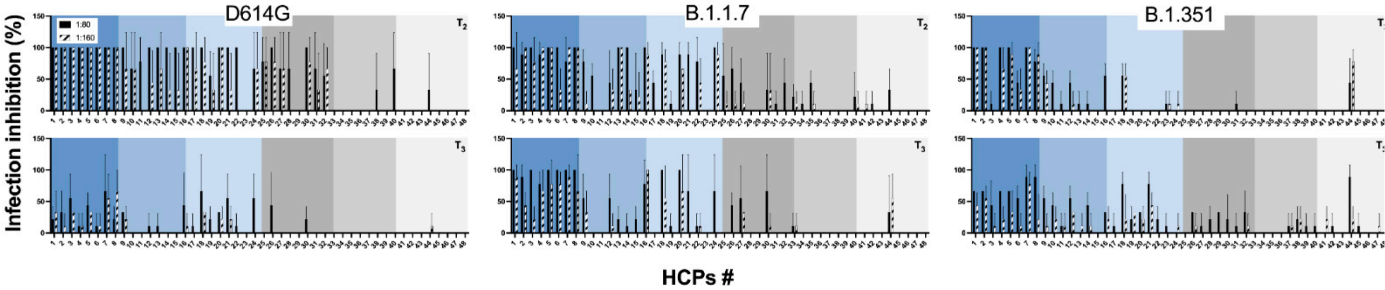

Figure S2. Neutralization assay. Neutralizing activity was assessed using two sera dilutions (1:80 and 1:160) against 0.01 MOI of three SARS-CoV-2 variants: D614G, B.1.1.7, B.1.351. Mean values + SD are reported, each condition was tested in triplicate. Sera are ordered according to the anti-RBD titer at T_1: dark blue, >2500 U/mL; blue, 2500 - 500 U/mL; light blue, 500 – 50 U/mL; dark grey 50 - 10 U/mL; light gray 10 - 1 U/mL, white < 1 U/mL.
